# Immunization error in Primary Health Care: an expression of the precariousness of nursing work

**DOI:** 10.1590/0034-7167-2025-0587

**Published:** 2026-09-28

**Authors:** Ferla Maria Simas Bastos Cirino, Gabriela Gonçalves Amaral, Sheila Aparecida Ferreira Lachtim, Anna Luiza de Fátima Pinho Lins Gryschek, Lucia Yasuko Izumi Nichiata

**Affiliations:** I Universidade de São Paulo. São Paulo, São Paulo, Brazil

**Keywords:** Primary Health Care, Immunization, Medication Errors, Workload, Nursing, Atención Primaria de Salud, Inmunización, Errores de Medicación, Carga de Trabajo, Enfermería.

## Abstract

**Objectives:**

to discuss immunization errors from the perspective of the nursing professionals’ work process in order to minimize their occurrence.

**Methods:**

this is a reflective study, grounded in concepts and constructs of nursing in public health.

**Results:**

the vaccination room is considered a strategic space in nursing practice, requiring both technical and managerial skills from the nurse. Overwork and precarious working conditions are associated with lower engagement in safe practices. Error prevention demands a systemic approach that goes beyond individual accountability and considers the organizational conditions that influence practice.

**Final Considerations:**

immunization errors cannot be reduced to technical or individual failures, but must be understood as an expression of the burnout experienced by nursing professionals subjected to precarious and unequal working conditions. Overcoming this requires strengthening autonomy, valuing dignified working conditions, and creating spaces for critical reflection.

**Descriptors:**

Primary Health Care; Immunization; Medication Errors; Workload; Nursing.

## RESUMEN

**Objetivos:**

analizar los errores de inmunización desde la perspectiva del proceso de trabajo de los profesionales de enfermería para minimizar su incidencia.

**Métodos:**

estudio reflexivo basado en conceptos y constructos de enfermería de salud pública.

**Resultados:**

la sala de vacunación se considera un espacio estratégico en la práctica enfermera, que requiere la intervención tanto técnica como gerencial del profesional de enfermería. La sobrecarga laboral y las condiciones laborales precarias se asocian con una menor participación en prácticas seguras. La prevención de errores requiere un enfoque sistémico que trascienda la responsabilidad individual y considere las condiciones organizacionales que influyen en la práctica.

**Consideraciones Finales:**

los errores de inmunización no pueden reducirse a fallas técnicas o individuales, sino que deben entenderse como una expresión del agotamiento que experimentan los profesionales de enfermería sometidos a condiciones laborales precarias y desiguales. Superarlo requiere fortalecer la autonomía, valorar las condiciones laborales dignas y crear espacios de reflexión crítica.

## INTRODUCTION

Vaccination is a strategic and essential activity in the nursing work process within Primary Health Care (PHC), in which nurses play a central, technically and scientifically sound, and legally supported role^(1)^. According to Resolution 795/2025 of the Federal Nursing Council, nurses, as coordinators of the nursing staff, are responsible for planning, supervising, and assessing vaccination actions, as well as ensuring adequate operating conditions in a vaccination room. These responsibilities include planning and adapting the physical infrastructure, maintaining, preparing, safely handling, and administering immunobiological agents, as well as monitoring vaccinated individuals after vaccination, ensuring the quality, safety, and effectiveness of the actions carried out by the staff^(1)^.

In this complex scenario, a vaccination room is a critical environment for immunization errors, preventable adverse events that compromise patient safety. Such failures are frequently associated with professional practice, especially due to the failure to strictly follow established technical protocols for the administration of immunobiological agents, a situation exacerbated by the complexity of the procedures involved and the need for continuous attention from the nursing staff^(2,3)^. Its occurrence may be associated with multiple stages of the process, including prescription, dispensing, preparation, administration, monitoring, transport, and storage^(2)^.

Among the factors associated with immunization errors, the following stand out: psychological factors (distraction, stress, and job dissatisfaction); deficiencies in continuing education; overload in work schedules; complexity of vaccination schedules; deficiencies related to knowledge and skills (regarding immunization and biosafety); inadequate infrastructure conditions at vaccination sites (noise, physical space); improper storage; and similarity between packaging of medications, vaccines, and professional fatigue^(4)^.

Frequently associated with nursing professionals working in vaccination rooms, the occurrence of errors and their notification invariably fall on the nursing staff, which can lead to underreporting of incidents, even out of fear of reprisal^(3,5)^. Since it is a passive surveillance system, underreporting may also be associated with its non-mandatory nature, resulting in an underestimation of the actual occurrence of errors and events supposedly attributable to vaccination or immunization (ESAVI)^(4,5)^.

Despite their low prevalence, they can, individually or in combination, compromise the effectiveness of immunization, causing a reduction or absence of the expected response, as well as potentially serious ESAVI, including fatal outcomes^(2)^.

Both immunization errors and associated ESAVI can lead to distrust in immunization programs. Given the current context, where there is an intensification in the dissemination of misinformation related to ESAVI, which fosters the proliferation of conspiracy theories and fuels the anti-vaccine movement, the occurrence of errors can act as a determining factor, increasing vaccine hesitancy and negatively impacting vaccination coverage^(6)^.

Although the literature on patient safety recognizes the systemic nature of error^(7)^, such approaches tend to remain at the immediate organizational level, without delving into the historical and social determinants of work in health.

This study invites reflection on the possibility that errors may be associated with burnout processes experienced by professionals in healthcare services, particularly in the context of vaccination rooms. The nursing staff’s work in vaccination is influenced by social relations and contradictions inherent to nursing as a social practice. A vaccination room is organized within the framework of production relations that can, in different contexts, both generate burnout and favor processes of professional strengthening^(8)^. Wear and tear occurs when production relations are exploitative and oppressive, leading to alienation and devaluation, and even resulting in immunization error. Strengthening occurs when production relations are more egalitarian and promote human development and collaboration^(8)^. Therefore, they can minimize the occurrence of these errors.

Presenting arguments about immunization errors requires a critical analysis of the work process. In this context, error will be analyzed as a socially produced phenomenon, not merely a technical one. The following guiding question is formulated: how can immunization errors in a vaccination room be minimized, considering the nursing professionals’ work process?

## OBJECTIVES

To discuss immunization errors from the perspective of the nursing professionals’ work process in order to minimize their occurrence.

## METHODS

This is a reflective study, based on concepts and constructs of nursing in public health^(9)^, specifically referring to the scope of work carried out in PHC, in the work process in vaccination rooms, anchored in theoretical reflection on the potential strains and strengths associated with this work process^(8)^.

These are the central points of a thesis entitled “*Erro de imunização na administração da vacina: perspectiva da gestão*”, by Dr. Núbia Virgínia D’Ávila Limeira de Araújo^(3)^, distinguished manager of the Immunization Division of the São Paulo State Health Department and professor in the Department of Collective Health Nursing, who passed away in 2024. Furthermore, in discussing the topic, the reflection relies on relevant international and national literature, incorporating inferences from the field of Latin American public health^(10)^.

The reflections were organized based on the definition of thematic axes constructed from a critical reading of scientific literature. These axes guided the development of the reflections, allowing for the articulation between theoretical framework, context of analysis, and interpretative critique.

## RESULTS

### Workflow in vaccination rooms

In nursing practice, a vaccination room is understood as a strategic and priority space for care, in which the nursing staff plays a fundamental role in the organization and execution of immunization actions in PHC.

The nursing staff’s responsibilities in vaccination rooms include storing and administering immunobiological agents^(1)^. Nurses are responsible for supervising and training the staff, and monitoring vaccination coverage indicators, ensuring compliance with the Brazilian National Immunization Program (In Portuguese, *Programa Nacional de Imunizações* - PNI) protocols and standards. Their role is strategic in strengthening health surveillance and expanding access to vaccination^(1,2)^. In this regard, there is a clear overlap of activities of different natures, caregiving and managerial, promoting a grueling work process^(8,11)^, which can lead to error^(12)^.

Given the multiplicity of responsibilities in PHC, nurses’ roles in vaccination rooms become reduced or even absent, leading to the delegation of responsibilities and functions that are exclusively the responsibility of nurses to nursing technicians. This overload can result in failures in staff training, surveillance of ESAVI, and ensuring strict compliance with PNI protocols and standards. Thus, vaccination room supervision, which requires continuous attention and technical updating, ends up being compromised, jeopardizing the safety of the vaccination process and the effectiveness of preventive actions in the territory^(13)^.

From a public health perspective, burnout is not limited to individual fatigue, but rather reflects the historical organization of the work process, characterized by intensification, fragmentation, and asymmetry between demands and available resources^(10)^.

Immunization errors can be associated with a lack of adequate training for nursing professionals and work overload. Staff turnover is also considered a contributing factor to increased immunization errors, as it hinders the consolidation of routines and adequate training for maintaining technical quality, especially in a context of the complexity of the vaccination schedule^(4)^.

A systematic review with meta-analysis showed that immunization errors result from partial or total non-compliance with established norms and protocols, the most common being administering the vaccine at the incorrect dosage, administering it outside the recommended age range, or an inadequate interval between doses^(4)^. A study that analyzed errors in the state of São Paulo, from 2015 to 2019, identified an error incidence rate of 5.3 per 100,000 doses administered, and among 1,595 notifications analyzed, the most prevalent error was the administration of vaccines outside the recommended age range, related to non-compliance with the vaccination schedule^(3)^. Through the vaccination record assessment method, a household survey revealed a prevalence of immunization errors ten times higher than that reported in the information systems during the same period, demonstrating the magnitude of underreporting of these errors^(5)^.

In addition to the visible increase in workload in PHC, with forms of work organization permeated by privatizing and productivist logic, which impose quantitative goals over quality, there are also inadequate working conditions, situations of violence and professional devaluation, which contribute to burnout and increase the likelihood of errors^(8)^, jeopardizing user and professional safety. Immunization errors are a current concern in public patient safety policies, highlighting flaws in healthcare work processes, and are linked to the field of occupational health by revealing the influence of working conditions and organization on the occurrence of preventable events.

In light of the report “To Err Is Human”, a conceptual framework for patient safety, and from the perspective of the “Swiss cheese” model based on Reason^(7)^, the vaccination process can be understood as a system susceptible to failures distributed across different levels, ranging from management and supervision to working conditions and unsafe acts. Thus, a systemic analysis of failures is important, shifting the focus from individual error to the organizational conditions that produce it.

Hence, organizational culture, within the context of the adopted healthcare model, directly reflects how care is conceived and practiced. In its broadest sense, the work structure can both favour and mitigate the occurrence of immunization errors^(3)^. It is recognized that the provision of healthcare is subject to errors, with the human factor being only one of the elements involved^(7)^. Therefore, preventing and mitigating errors requires a systemic approach that goes beyond individual accountability and considers the organizational conditions that influence daily practice^(3)^.

In this context, strategies focused exclusively on training, supervision, and the adequate provision of resources may be insufficient, especially when based on the notion that immunization errors stem solely from professionals’ individual actions. Such an approach can contribute to underreporting of events and fear of institutional reprisals. Thus, the analysis of immunization errors should shift the focus from isolated causes to understanding the organizational contexts that enable them^(3)^.

Although the hierarchical nature of PNI is understandable, given the need for coordination and clear definition of responsibilities among governmental bodies, it is fundamental to analyze how this policy has been effectively implemented in the territories. This implies considering not only the organization of healthcare services from an individual, biological, and behavioral perspective, but also recognizing that multiple social, cultural, and territorial dimensions influence the health-disease process and condition the effectiveness of actions in each locality^(3)^.

### Strengthening nursing professionals to minimize the occurrence of immunization errors

It is essential to promote collective spaces for reflection so that professionals can rethink work processes and collectively build strategies to address immunization errors. Furthermore, nurses’ active participation in service planning and management strengthens their autonomy, provides clarity on care flows, and increases their commitment to practice safety^(12)^.

Improving nursing professionals’ working conditions is fundamental to reducing errors in the vaccination process, as well as increasing their confidence in vaccines and, consequently, promoting safe and proactive behaviors that ensure user safety. A broad assessment of French nurses demonstrated that dissatisfaction with working conditions is associated with lower engagement in safe practices, including an impact on lower vaccination recommendations for users of healthcare services^(14)^. Studies^(3,4)^ indicate that having well-defined processes, adequate organization, and institutional support is strategic for preventing failures during vaccine preparation and administration. It is also important to have clear protocols, continuous training, and active review of processes to minimize errors and ensure safety^(3,4)^.

The absence of nurses in vaccination rooms reflects the organization of the work process in PHC, with overlapping responsibilities. This situation not only prevents them from being present during immunization but also depletes their productive capacity and physical health. This process of intensified and overloaded work, coupled with a tense workflow, generates gradual harm to workers and the care process^(15)^.

Practical actions, such as reviewing staffing levels based on the activities currently performed by professionals, constitute a strategy to minimize the occurrence of errors. It is obvious that the mere presence of a nurse does not, by itself, guarantee the elimination of immunization errors. Therefore, it needs to be combined with professional development, including improved working conditions, reduced working hours, structured career paths, and contractual stability, which can contribute to reducing turnover and directly impacting quality of care^(3,8)^.

It is necessary to consider that immunization errors, as occurrences in healthcare work, should be analyzed as a direct consequence of the burnout experienced by professionals working in PHC. Historically, the precariousness of work has been exacerbated by the flexibilization of labor laws, especially after the adoption of a neoliberal perspective by the Brazilian state, and worsened with the Fiscal Responsibility Law, which dismantles worker protections and reduces the number of public service examinations, resulting in insufficient hiring to meet healthcare needs. The overload and contractual instability observed in the daily practice of nursing are not exceptions, but rather express structural trends of precarious work in healthcare, associated with neoliberal reforms and the flexibilization of labor relations in the public sector^(15)^.

Nursing is directly impacted by this process of productive restructuring, evidenced by the increase in unemployment rates, the retraction of social and labor rights, and the intensification of pauperization and precariousness of labor relations^(8)^. In this context, immunization errors should not be interpreted merely as individual technical failures, but as a concrete manifestation of adverse working conditions, revealing professionals’ illness and the degradation of healthcare practices, especially in PHC, where professional burnout is more acutely expressed^(3)^.

In this context, the authors call for a deeper understanding of immunization error, from the perspective of the ethical-political commitment to strengthening nursing professionals in PHC, as highlighted in the study that underpinned this reflection^(3)^, going beyond the technical analysis of the error.

#### Study limitations

It is recognized that this is a theoretical reflection anchored in the frameworks of public health nursing and in the authors’ professional and academic trajectory, which gives the text an interpretative and subjective character.

#### Contributions to nursing, health, or public policy

This reflection contributes to the field by discussing immunization errors from the perspective of strengthening nursing professionals, highlighting the need to understand it as a multifactorial phenomenon, not just an individual one. The reflection shows that error prevention is intrinsically linked to improving working conditions, professional recognition, and the presence of supportive supervision. In this context, it is imperative to advocate, as a political and organizational guideline, the pressing need for a dedicated nurse in vaccination rooms, considering the growing number of vaccines available, with different characteristics, dosages, routes of administration, and vaccination schedules. The presence of this professional is essential to ensure technical quality, user safety, and the effectiveness of vaccination actions, constituting a strategic element for consolidating a safety culture and reducing avoidable errors.

Therefore, it is recommended that the exclusive presence of a nurse in the vaccination room be recognized as a structuring and indispensable component of patient safety policy and the quality of vaccination care. This guideline should guide the organization of services and the planning of actions at all management levels of the Brazilian Health System (In Portuguese, *Sistema Único de Saúde* - SUS). Ensuring adequate organizational conditions for nurses to act as the direct technical supervisors of the immunization process contributes to strengthening the safety culture, valuing nurses’ work, and ensuring the PNI’s sustainability within the scope of PHC.

## FINAL CONSIDERATIONS

Immunization errors cannot be reduced to technical or individual failures, but must be understood as an expression of the strain on nursing professionals subjected to precarious and unequal working conditions. The normalization of overload, contractual instability, and insufficient institutional resources reflects a broader process of precariousness and flexibilization of labor relations, intensified in recent decades under neoliberal logic.

In this regard, questioning error also means questioning the model of work management in healthcare, which tends to hold the individual responsible over a structural and collective analysis. Overcoming this reductionism requires strengthening nursing’s professional autonomy, valuing dignified working conditions, and creating spaces for critical reflection on care processes.

Recognizing immunization error as a socially determined phenomenon implies shifting it from the moral and individual field to the ethical-political plane of the organization of work in health, reaffirming the centrality of nursing and the defense of the SUS as a universal public policy.

## Data Availability

Not applicable.
