# Supplementary material for: Immunization error in Primary Health Care: an expression of the precariousness of nursing work
Source: Rev Bras Enferm. 2026 Sep 28;79:e20250587. doi: 10.1590/0034-7167-2025-0587 (PMC13623391; doi:10.1590/0034-7167-2025-0587)
Supplement: Translation

# Translation: Erro de imunização na Atenção Primária à Saúde: expressão da precarização do trabalho em enfermagem

**DOI:** 10.1590/0034-7167-2025-0587pt

**Authors:** Ferla Maria Simas Bastos Cirino, Gabriela Gonçalves Amaral, Sheila Aparecida Ferreira Lachtim, Anna Luiza de Fátima Pinho Lins Gryschek, Lucia Yasuko Izumi Nichiata

**Affiliations:** I Universidade de São Paulo. São Paulo, São Paulo, Brasil

**Keywords:** Atenção Primária à, Saúde, Imunização, Erros de Medicação, Carga de Trabalho, Enfermagem.

## Abstract

**Objetivos:**

discutir o erro de imunização na perspectiva do processo de trabalho dos profissionais de enfermagem para minimizar a sua ocorrência.

**Métodos:**

estudo de natureza reflexiva, fundamentado em conceitos e construtos da enfermagem em saúde coletiva.

**Resultados:**

a sala de vacinação é considerada um espaço estratégico na prática da enfermagem, exigindo atuação técnica e gerencial do enfermeiro. A sobrecarga e a precarização do trabalho estão associadas ao menor engajamento em práticas seguras. A prevenção de erros demanda abordagem sistêmica que ultrapasse a responsabilização individual e considere as condições organizacionais que influenciam a prática.

**Considerações Finais:**

o erro de imunização não pode ser reduzido a falhas técnicas ou individuais, mas deve ser entendido como expressão do desgaste do profissional de enfermagem submetido a condições precárias e desiguais de trabalho. Superá-lo exige fortalecer a autonomia, valorizar as condições dignas de trabalho e criar espaços de reflexão crítica.

## INTRODUÇÃO

A vacinação constitui uma atividade estratégica e essencial no processo de trabalho da enfermagem no âmbito da Atenção Primária à Saúde (APS), na qual o enfermeiro exerce papel central, técnico-científico e legalmente respaldado^(1)^. De acordo com a Resolução Conselho Federal de Enfermagem nº 795/2025, compete ao enfermeiro, enquanto coordenador da equipe de enfermagem, planejar, supervisionar e avaliar as ações de vacinação, bem como garantir as condições adequadas de funcionamento da sala de vacinação. Essas atribuições incluem o planejamento e a adequação da infraestrutura física, a conservação, o preparo, o manejo seguro e a administração de imunobiológicos, bem como o monitoramento pós-vacinal dos indivíduos vacinados, garantindo a qualidade, a segurança e a efetividade das ações desenvolvidas pela equipe^(1)^.

Nesse cenário complexo, a sala de vacinação é um ambiente crítico para a ocorrência de erros de imunização, eventos adversos evitáveis que comprometem a segurança do paciente. Tais falhas estão frequentemente associadas à prática profissional, sobretudo pelo não seguimento rigoroso dos protocolos técnicos estabelecidos para a administração de imunobiológicos, o que se agrava diante da complexidade dos procedimentos envolvidos e da exigência de atenção contínua por parte da equipe de enfermagem^(2,3)^. Sua ocorrência pode estar associada às múltiplas etapas do processo, incluindo prescrição, dispensação, preparação, administração, monitoramento, transporte e armazenamento^(2)^.

Entre os condicionantes associados aos erros de imunização, destacam-se aqueles ligados a: fator psicológico (distração, estresse, e insatisfação com o trabalho); deficiência na educação continuada; sobrecarga nas escalas de trabalho; complexidade dos esquemas vacinais; deficiências ligadas ao conhecimento e competências (sobre imunização e biossegurança); condições inadequadas da infraestrutura dos locais de vacinação (ruído, espaço físico); armazenamento impróprio; e semelhança entre embalagens de medicamentos, vacinas e fadiga dos profissionais^(4)^.

Frequentemente atrelados aos profissionais de enfermagem, atuantes na sala de vacinação, a ocorrência do erro e a notificação invariavelmente recaem sobre a equipe de enfermagem, o que pode gerar subnotificação das ocorrências, até por medo de represália^(3,5)^. Por se tratar de um sistema de vigilância passiva, a subnotificação também pode estar associada à sua não obrigatoriedade, ocasionando em subestimação da real ocorrência dos erros e dos eventos supostamente atribuíveis à vacinação ou imunização (ESAVI)^(4,5)^.

Apesar da baixa prevalência, podem, isoladamente ou em associação, comprometer a eficácia da imunização, ocasionando redução ou ausência da resposta esperada, além de potenciais ESAVI graves, incluindo desfechos fatais^(2)^.

Tanto os erros de imunização quanto os ESAVI associados podem acarretar desconfiança nos programas de imunização. Diante do atual contexto, onde se observa uma intensificação na disseminação de desinformação relacionada aos ESAVI, que propicia a proliferação de teorias da conspiração e fomenta o movimento antivacina, a ocorrência de erros pode atuar como fator determinante, ampliando a hesitação vacinal e impactando negativamente as coberturas vacinais^(6)^.

Embora a literatura sobre segurança do paciente reconheça o caráter sistêmico do erro^(7)^, tais abordagens tendem a permanecer no nível organizacional imediato, sem aprofundar as determinações históricas e sociais do trabalho em saúde.

O presente estudo convida à reflexão de que os erros podem estar associados a processos de desgaste vivenciados pelos profissionais nos serviços de saúde, particularmente no contexto das salas de vacinação. O trabalho da equipe de enfermagem na vacinação é influenciado por relações sociais e por contradições próprias da enfermagem enquanto prática social, sendo a sala de vacinação organizada no âmbito de relações de produção que podem, em diferentes contextos, tanto gerar desgaste quanto favorecer processos de fortalecimento profissional^(8)^. O desgaste ocorre quando as relações de produção são exploradoras e opressoras, levando à alienação e à desvalorização, resultando, inclusive, no erro de imunização. O fortalecimento ocorre quando as relações de produção são mais igualitárias e promovem o desenvolvimento humano e a colaboração^(8)^. Portanto, podem minimizar a ocorrência desses erros.

A exposição dos argumentos sobre os erros de imunização exige uma análise crítica do processo de trabalho. Nesse contexto, o erro será analisado como fenômeno socialmente produzido, não apenas técnico. Formula-se a seguinte questão norteadora: de que maneira pode-se minimizar os erros de imunização na sala de vacinação, ao considerar o processo de trabalho dos profissionais de enfermagem?

## OBJETIVOS

Discutir o erro de imunização na perspectiva do processo de trabalho dos profissionais de enfermagem para minimizar a sua ocorrência.

## MÉTODOS

Trata-se de estudo de natureza reflexiva, fundamentado em conceitos e construtos da enfermagem em saúde coletiva^(9)^, especificamente referido ao âmbito do trabalho realizado na APS, no processo de trabalho em salas de vacinação, ancorado na reflexão teórica acerca dos potenciais desgastes e fortalecimentos associados a esse processo laboral^(8)^.

Esses são os pontos centrais da tese intitulada “Erro de imunização na administração da vacina: perspectiva da gestão”, da Dr^a^. Núbia Virgínia D’Ávila Limeira de Araújo^(3)^, ilustre gestora da Divisão de Imunização da Secretaria de Estado da Saúde de São Paulo e docente do Departamento de Enfermagem em Saúde Coletiva, falecida em 2024. Além disso, ao discorrer sobre a temática, a reflexão se apoia na literatura internacional e nacional pertinente, incorporando inferências oriundas do campo da saúde coletiva latino-americana^(10)^.

As reflexões foram organizadas a partir da definição de eixos temáticos, construídos com base na leitura crítica da literatura científica. Esses eixos orientaram o desenvolvimento das reflexões, permitindo a articulação entre referencial teórico, contexto de análise e crítica interpretativa.

## RESULTADOS

### O processo de trabalho na sala de vacinação

Na prática da enfermagem, a sala de vacinação é compreendida como um espaço estratégico e prioritário de cuidado, no qual a equipe de enfermagem assume papel fundamental na organização e execução das ações de imunização na APS.

Entre as atribuições da equipe de enfermagem na sala de vacinação, estão a conservação e a administração dos imunobiológicos^(1)^. O enfermeiro é responsável pela supervisão e capacitação da equipe, e pelo monitoramento dos indicadores de cobertura vacinal, assegurando o cumprimento dos protocolos e normas do Programa Nacional de Imunizações (PNI). Sua atuação é estratégica para o fortalecimento da vigilância em saúde e para a ampliação do acesso à vacinação^(1,2)^. Nesse sentido, há uma clara sobreposição de atividades de diferentes naturezas, assistencial e gerencial, promovendo um processo de trabalho desgastante^(8,11)^, que pode induzir ao erro^(12)^.

Diante da multiplicidade de atribuições na APS, a atuação do enfermeiro na sala de vacinação se torna reduzida ou até mesmo ausente, o que acaba por levar à delegação de responsabilidades e funções, que são exclusivas do enfermeiro, para os técnicos de enfermagem. Essa sobrecarga pode resultar em falhas na capacitação da equipe, na vigilância dos ESAVI e na garantia do cumprimento rigoroso dos protocolos e normas do PNI. Assim, a supervisão da sala de vacinação, que exige contínua atenção e atualização técnica, acaba sendo prejudicada, colocando em risco a segurança do processo de vacinação e a efetividade das ações preventivas no território^(13)^.

Na perspectiva da saúde coletiva, o desgaste não se reduz à fadiga individual, mas expressa a forma histórica de organização do processo de trabalho, marcada por intensificação, fragmentação e assimetria entre exigências e meios disponíveis^(10)^.

Os erros de imunização podem estar associados à falta de capacitação adequada dos profissionais de enfermagem e à sobrecarga de trabalho. A rotatividade de profissionais também é considerada um condicionante para o aumento de erros de imunização, por dificultar a consolidação das rotinas e da capacitação adequada para a manutenção da qualidade técnica, especialmente em um cenário de complexidade do calendário vacinal^(4)^.

Revisão sistemática com metanálise evidenciou que os erros de imunização são resultantes do descumprimento parcial ou total de normas e protocolos estabelecidos, sendo os mais comuns administração da vacina na dosagem incorreta, aplicação fora da idade recomendada ou intervalo inadequado entre as doses^(4)^. Estudo que analisou os erros no estado de São Paulo, no período de 2015 a 2019, identificou taxa de incidência de erros de 5,3 por 100.000 doses aplicadas. Entre as 1.595 notificações analisadas, o erro mais prevalente foi a administração de vacina fora de idade, referente ao descumprimento do calendário vacinal^(3)^. Por meio do método de avaliação de cadernetas de vacinação, um inquérito domiciliar evidenciou uma prevalência de erros de imunização dez vezes maior que a notificada nos sistemas de informação no mesmo período, demonstrando a magnitude da subnotificação desses erros^(5)^.

Além do aumento visível da carga de trabalho na APS, com formas de organização do trabalho impregnadas pela lógica privatista e produtivista, que impõem metas quantitativas em detrimento da qualidade, há também condições inadequadas de trabalho, situações de violência e desvalorização profissional, que contribuem para o desgaste e aumentam a probabilidade de falhas^(8)^, colocando em risco a segurança dos usuários e dos próprios profissionais. O erro de imunização integra as preocupações atuais das políticas públicas de segurança do paciente, ao evidenciar falhas nos processos de trabalho em saúde, articulando-se ao campo da saúde do trabalhador ao revelar a influência das condições e da organização do trabalho sobre a ocorrência de eventos evitáveis.

À luz do relatório “*To Err Is Human*”, marco conceitual da segurança do paciente, e sob a perspectiva do modelo do “queijo suíço” fundamentado por Reason^(7)^, o processo de vacinação pode ser compreendido como um sistema suscetível a falhas distribuídas em diferentes níveis, que vão desde a gestão e a supervisão até as condições de trabalho e os atos inseguros. Assim, é importante que haja uma análise sistêmica sobre as falhas, deslocando o foco do erro individual para as condições organizacionais que o produzem.

Nesse sentido, a cultura organizacional, no contexto do modelo de saúde adotado, reflete diretamente a forma como o cuidado é concebido e praticado. A estrutura do trabalho, em sua dimensão ampliada, pode tanto favorecer quanto mitigar a ocorrência de erros de imunização^(3)^. Reconhece-se que a prestação do cuidado em saúde está sujeita a falhas, sendo o fator humano apenas um dos elementos envolvidos^(7)^. Assim, a prevenção e a mitigação de erros demandam uma abordagem sistêmica que ultrapasse a responsabilização individual e considere as condições organizacionais que influenciam a prática cotidiana^(3)^.

Nesse contexto, estratégias centradas exclusivamente em capacitações, supervisões e no fornecimento adequado de insumos podem ser insuficientes, especialmente quando baseadas na concepção de que o erro de imunização decorre unicamente da ação individual dos profissionais. Tal abordagem pode contribuir para a subnotificação dos eventos e para o temor de represálias institucionais. Assim, a análise do erro de imunização deve deslocar o foco da causa isolada para a compreensão dos contextos organizacionais que o possibilitam^(3)^.

Embora o caráter hierárquico do PNI seja compreensível, devido à necessidade de coordenação e definição clara das atribuições entre as instâncias governamentais, é fundamental analisar como essa política tem sido efetivamente implementada nos territórios. Isso implica considerar não apenas a organização dos serviços de saúde sob a ótica individual, biológica e comportamental, mas também reconhecer que as múltiplas dimensões, sociais, culturais e territoriais influenciam o processo de saúde-doença e condicionam a efetividade das ações em cada localidade^(3)^.

### Fortalecer os profissionais de enfermagem para minimizar ocorrência do erro de imunização

Faz-se primordial promover espaços coletivos de reflexão para que os profissionais (re)pensem os processos de trabalho e construam coletivamente estratégias de enfrentamento do erro de imunização. Além disso, a participação ativa dos enfermeiros no planejamento e na gestão dos serviços fortalece sua autonomia, confere clareza sobre os fluxos de atendimento e amplia o compromisso com a segurança das práticas^(12)^.

A melhoria das condições de trabalho dos profissionais de enfermagem é fundamental para reduzir erros no processo de vacinação, além de aumentar a confiança desses nas vacinas e, consequentemente, promover comportamentos seguros e proativos, que conferem a segurança do usuário. Avaliação ampla dos enfermeiros franceses demonstrou que a insatisfação com as condições de trabalho está associada a um menor engajamento em práticas seguras, inclusive com impacto na menor recomendação vacinal aos usuários dos serviços de saúde^(14)^. Estudos^(3,4)^ apontam que ter processos bem definidos, organização adequada e suporte institucional é estratégico para prevenir falhas durante o preparo e administração de vacinas. É importante também ter protocolos claros, treinamento contínuo e revisão ativa dos processos para minimizar erros e garantir a segurança^(3,4)^.

A ausência do enfermeiro na sala de vacinação é reflexo da organização do processo de trabalho na APS, com sobreposição de atribuições. Tal situação não apenas o impede de estar presente na imunização, como também esgota suas forças produtivas e sua saúde física. Esse processo de intensificação e sobrecarga de trabalho, aliado ao fluxo tensionado, gera prejuízos gradativos aos trabalhadores e ao processo de cuidado^(15)^.

Ações práticas, como rever o dimensionamento de pessoal apoiado nas atividades realizadas hoje pelos profissionais, constituem uma estratégia para minimizar a ocorrência do erro. É óbvio que apenas a presença do enfermeiro não garanta, por si só, eliminar os erros de imunização. Portanto, precisa estar aliada à valorização profissional, inclusive apoiada em melhores condições de trabalho, com redução da jornada de trabalho, carreira estruturada e estabilidade contratual, que podem contribuir para a redução da rotatividade, impactando diretamente a qualidade da assistência^(3,8)^.

É preciso considerar que o erro de imunização, como ocorrência do trabalho em saúde, deve ser analisado como consequência direta do desgaste do profissional que atua na APS. Historicamente, a precarização do trabalho foi adensada com a flexibilização das leis trabalhistas, principalmente após a adoção da perspectiva neoliberal pelo Estado brasileiro, e se agravou com a Lei da Responsabilidade Fiscal, que desmantela a proteção dos trabalhadores e diminui a realização de concursos, resultando em contratações deficitárias para atender às necessidades de saúde. A sobrecarga e a instabilidade contratual observadas no cotidiano da enfermagem não constituem exceções, mas expressam tendências estruturais de precarização do trabalho em saúde associadas às reformas neoliberais e à flexibilização das relações laborais no setor público^(15)^.

A enfermagem é diretamente impactada por esse processo de reestruturação produtiva, evidenciado pelo aumento dos índices de desemprego, pela retração dos direitos sociais e trabalhistas, e pela intensificação da pauperização e da precarização das relações laborais^(8)^. Nesse contexto, o erro de imunização não deve ser interpretado apenas como uma falha técnica individual, mas como uma manifestação concreta das condições adversas de trabalho, revelando o adoecimento do profissional e a degradação das práticas de cuidado em saúde, especialmente na APS, onde o desgaste do profissional se expressa de forma mais aguda^(3)^.

Nesse contexto, as autoras propõem uma compreensão aprofundada do erro de imunização, considerando o compromisso ético-político com o fortalecimento dos profissionais de enfermagem na APS, conforme destaca o estudo balizador desta reflexão^(3)^, transcendendo a análise técnica do erro.

#### Limitações do estudo

Reconhece-se que se trata de reflexão teórica, ancorada em referenciais da enfermagem em saúde coletiva e na trajetória profissional e acadêmica das autoras, o que imprime ao texto um caráter interpretativo e subjetivo.

#### Contribuições para a área da enfermagem, saúde ou políticas públicas

A reflexão contribui para a área, ao discutir o erro de imunização sob a perspectiva do fortalecimento dos profissionais de enfermagem, destacando a necessidade de compreendê-lo como um fenômeno multifatorial, não apenas individual. A reflexão evidencia que a prevenção de erros está intrinsecamente relacionada ao aprimoramento das condições de trabalho, à valorização profissional e à presença de uma supervisão apoiadora. Nesse contexto, é imperioso defender, como diretriz política e organizacional, a necessidade premente de um enfermeiro exclusivo na sala de vacinação, considerando o crescente número de vacinas disponíveis, com diferentes características, posologias, vias de administração e esquemas vacinais. A presença desse profissional é condição essencial para assegurar a qualidade técnica, a segurança dos usuários e a efetividade das ações de vacinação, constituindo elemento estratégico para a consolidação da cultura de segurança e a redução de erros evitáveis.

Diante disso, recomenda-se o reconhecimento da presença exclusiva de um enfermeiro na sala de vacinação como componente estruturante e indispensável da política de segurança do paciente e da qualidade da assistência em vacinação. Essa diretriz deve orientar a organização dos serviços e o planejamento das ações em todos os níveis de gestão do Sistema Único de Saúde (SUS). A garantia de condições organizacionais adequadas à atuação do enfermeiro como responsável técnico direto pelo processo de imunização contribui para o fortalecimento da cultura de segurança, a valorização do trabalho da enfermagem e a sustentabilidade do PNI no âmbito da APS.

## CONSIDERAÇÕES FINAIS

O erro de imunização não pode ser reduzido a falhas técnicas ou individuais, mas deve ser entendido como expressão do desgaste do profissional de enfermagem submetido a condições precárias e desiguais de trabalho. A naturalização da sobrecarga, da instabilidade contratual e da insuficiência de recursos institucionais reflete um processo mais amplo de precarização e flexibilização das relações laborais, intensificado nas últimas décadas sob a lógica neoliberal.

Nesse sentido, problematizar o erro é também problematizar o modelo de gestão do trabalho em saúde, que tende a responsabilizar o indivíduo em detrimento de uma análise estrutural e coletiva. A superação desse reducionismo exige o fortalecimento da autonomia profissional da enfermagem, a valorização das condições dignas de trabalho e a criação de espaços de reflexão crítica sobre os processos de cuidado.

Reconhecer o erro de imunização como fenômeno socialmente determinado implica deslocá-lo do campo moral e individual para o plano ético-político da organização do trabalho em saúde, reafirmando a centralidade da enfermagem e a defesa do SUS como política pública universal.

## Data Availability

Não se aplica.
